# A Nucleotide Sugar Transporter Involved in Glycosylation of the *Toxoplasma* Tissue Cyst Wall Is Required for Efficient Persistence of Bradyzoites

**DOI:** 10.1371/journal.ppat.1003331

**Published:** 2013-05-02

**Authors:** Carolina E. Caffaro, Anita A. Koshy, Li Liu, Gusti M. Zeiner, Carlos B. Hirschberg, John C. Boothroyd

**Affiliations:** 1 Department of Microbiology & Immunology, Stanford University School of Medicine, Stanford, California, United States of America; 2 Department of Medicine (Infectious Diseases), Stanford University School of Medicine, Stanford, California, United States of America; 3 Department of Molecular and Cell Biology, Boston University Goldman School of Dental Medicine, Boston, Massachusetts, United States of America; University of Georgia, United States of America

## Abstract

*Toxoplasma gondii* is an intracellular parasite that transitions from acute infection to a chronic infective state in its intermediate host *via* encystation, which enables the parasite to evade immune detection and clearance. It is widely accepted that the tissue cyst perimeter is highly and specifically decorated with glycan modifications; however, the role of these modifications in the establishment and persistence of chronic infection has not been investigated. Here we identify and biochemically and biologically characterize a *Toxoplasma* nucleotide-sugar transporter (TgNST1) that is required for cyst wall glycosylation. *Toxoplasma* strains deleted for the *TgNST1* gene (Δ*nst1*) form cyst-like structures *in vitro* but no longer interact with lectins, suggesting that Δ*nst1* strains are deficient in the transport and use of sugars for the biosynthesis of cyst-wall structures. *In vivo* infection experiments demonstrate that the lack of TgNST1 activity does not detectably impact the acute (tachyzoite) stages of an infection or tropism of the parasite for the brain but that Δ*nst1* parasites are severely defective in persistence during the chronic stages of the infection. These results demonstrate for the first time the critical role of parasite glycoconjugates in the persistence of *Toxoplasma* tissue cysts.

## Author Summary

The *Toxoplasma* tissue cyst is essential to the persistence of the parasite during the chronic infection of an immunocompetent host. While significant efforts have been made to identify molecular factors that trigger and sustain parasite encystation, the role of the glycoconjugates that decorate the cyst wall has received little attention. Here we identify and characterize a *bona fide* nucleotide-sugar transporter, TgNST1, whose activity is required for the proper assembly of cyst wall glycoconjugates. We found that deletion of TgNST1 interferes with glycosylation during both the tachyzoite and bradyzoite stages of infection, and we observed substantial defects in the ability of *Δnst1* parasites to maintain chronic infection. Surprisingly, *Δnst1* parasites were not significantly defective in acute infection of mice, and showed wild type levels and migration rates to the brain. These results highlight the important role of cyst-wall glycosylation in parasite persistence during chronic infection, and suggest that drugs targeting nucleotide-sugar transporters and other enzymes required for glycosylation, perhaps in combination with drugs targeting other pathways, might be useful to prevent the establishment of chronic parasite infection.

## Introduction

The Apicomplexa are a phylum of protozoan organisms, many of which are pathogens with human and veterinary relevance. The Apicomplexan parasites share the remarkable ability to invade and propagate within host cells. Among this group, *Toxoplasma gondii* is an exemplar of the ability to undergo efficient transmission and persist within immunocompetent hosts without causing significant disease [1]. Furthermore, while the complex life cycle of these parasites includes sexual and asexual stages, *Toxoplasma* has the unusual capacity to circumvent the sexual stage and clonally disseminate through intermediate hosts [2]. Key to this clonal proliferation is *Toxoplasma's* ability to interconvert between a rapidly proliferating tachyzoite and a slow-dividing form, the bradyzoite, which encysts within host cells. The bradyzoite form is critical for the establishment of a chronic infection, persistence within the host and transmission to other hosts *via* ingestion of raw meat or other tissue from an infected animal. Under circumstances that cause immunosuppression, *Toxoplasma* bradyzoites that differentiate back to tachyzoites can reinitiate extensive rounds of proliferation causing significant disease [3], [4].

*Toxoplasma* tissue cysts appear to be nearly quiescent and much less immunogenic than tachyzoites, occupying a safe intracellular niche for the parasite. Tissue cysts can be observed in the brain of infected animals as early as 10 days postinfection [5]. While the number of cysts can remain high throughout the life of the host, some appear to occasionally rupture [6] and release parasites that could infect and encyst within neighboring host cells. *In vivo* tissue cysts can be 10–100 µm in diameter with a cyst wall that is 200–850 nm thick surrounding an osmiophilic matrix composed of granular material and small vesicles [7], [8]. Previous studies have shown that the lectins Dolichos Biflorus Agglutinin (DBA), which binds *N*-acetylgalactosamine (GalNAc) moieties, and succinylated Wheat Germ Agglutinin (sWGA), which binds to *N*-acetylglucosamine (GlcNAc) residues, effectively stain the cyst wall [9]. These lectins are commonly used as markers to identify *in vitro* and *in vivo Toxoplasma* cysts, although the specific parasite ligands bound by these lectins have not been fully characterized [5]. Previously, a mouse monoclonal antibody (MAb 73.18) was described that reacts with a cyst wall protein, CST1, by western blot and immunofluorescence [10]. A band of about the same size in Western Blots is also recognized by DBA, by sera of chronically infected animals and by a rat MAb CC2 [11]. Importantly, the MAb 73.18 no longer recognizes this protein when glycans are destroyed by sodium metaperiodate treatment suggesting that the MAb 73.18 might specifically recognize a glycoepitope on CST1 [10]. The identity and the role of the CST1 protein have not been described.

Recent studies have begun to link cyst wall glycans to cyst development. Craver *et al.* identified a gene encoding a protein with homology to a proteophosphoglycan from *Leishmania* that, when disrupted, impairs cyst wall formation and conversion from tachyzoites to bradyzoites [12]. As yet, however, it is not known if this protein is in fact glycosylated. Despite the mounting evidence linking the cyst wall to *Toxoplasma* persistence, the full repertoire of cyst wall glycoconjugates and their specific roles during infection have not yet been reported.

To address the role of *Toxoplasma* cyst wall glycans during infection, we are interested in proteins required for the early steps in glycoconjugate formation, the nucleotide-sugar transporters (NSTs). NSTs are polytopic proteins that localize to the endoplasmic reticulum (ER)/Golgi apparatus (GA) membrane and translocate nucleotide-sugars from the cytosol to the lumen of the organelle where they are transferred to the corresponding acceptors [13]. Here we show that TgNST1 is a *bona fide* NST that is capable of transporting UDP-GlcNAc and UDP-GalNAc across membranes. Knockout mutants and *in vivo* studies reveal a critical role for this transporter in the persistence of *Toxoplasma* tissue cysts in animals.

## Results

### Identification and *in vitro* characterization of the *Toxoplasma* nucleotide-sugar transporter TgNST1

To dissect the role of the cyst wall glycans in *Toxoplasma* persistence, we searched the *Toxoplasma* genome for known gene families required for early stages of glycoconjugate biosynthesis. We identified three *Toxoplasma* genes with substantial homology to known NSTs: *TgME49_067380*, *TgME49_054580* and *TgME49_067730*. To identify a gene whose deletion would be expected to impair glycosylation of cyst wall components, we compared these sequences to C03H5.2, an UDP-GalNAc/UDP-GlcNAc transporter from *C.elegans* that has the same substrate specificity as the lectins that bind to the wall of *Toxoplasma* tissue cysts [14]. One of the three genes, *TgME49_067380*, displayed 45% amino acid similarity to C03H5.2, versus 9% and 14% similarity for *TgME49_054580* and *TgME49_067730*, respectively (data not shown). We therefore selected *TgME49_067380* as likely key to the synthesis of the glycoconjugates in the walls of *Toxoplasma* tissue cysts and named it *TgNST1*.

**Figure 1:**
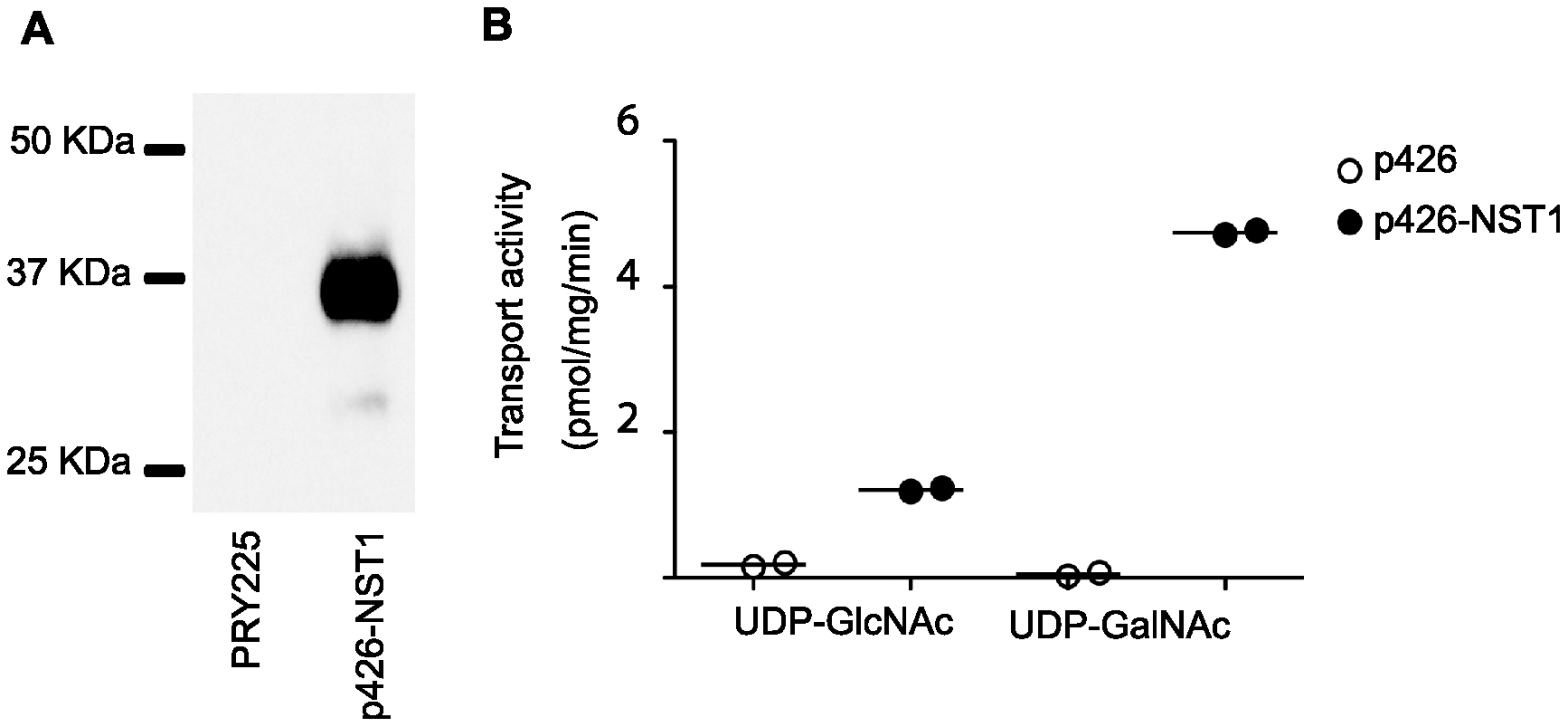
*Toxoplasma* TgNST1 is a nucleotide-sugar transporter that transports UDP-GlcNAc and UDP-GalNac. A) Analysis of protein expression by western blot. Total extracts from *Saccharomyces cerevisiae* strain PRY225 and the same strain transformed with the p426 vector encoding the *Toxoplasma* NST1HA protein (TgNST1) were resolved by SDS-PAGE followed by transfer to PVDF membrane. Detection of the recombinant protein was done using an anti-HA antibody. B) 1 mg of membrane vesicles from yeast transformed with an empty vector or the vector encoding the TgNST1HA protein were incubated with radioactive and unlabeled UDP-GlcNAc or UDP-GalNAc for 4 min. Final concentration of nucleotide-sugar is 2 µM per sample. Vesicles were separated by centrifugation and radioactivity within vesicles was measured in the pellet after acid precipitation. Transport activity is calculated as the difference of radioactive solutes in the vesicle pellet after incubation at 30°C vs 0°C and expressed as pmol of nucleotide sugar/mg protein/min.

To assess the NST activity and specificity of TgNST1 *in vitro*, we utilized a *Saccharomyces cerevisiae* microsome activity assay that takes advantage of the fact that yeast only possesses endogenous transport activity for GDP-Mannose and UDP-Glucose. First, we expressed the *TgNST1* coding region fused to a HA tag in *S. cerevisiae*. As shown in figure 1A, microsomes of *S. cerevisiae* transformed with the vector encoding the TgNST1 fusion protein express a protein of about the expected mobility (predicted size ∼42 KDa but polytopic proteins like TgNST1 are known to migrate somewhere faster than expected in SDS-PAGE [14]). We then isolated Golgi-enriched vesicles from yeast transformed with either the TgNST1-expressing vector or an empty vector, and determined the integrity of the resulting vesicles by measuring the activity of a lumenal Golgi protein, GDPase, in intact or detergent-permeabilized vesicles. Vesicle samples from the two strains with similarly high integrity (∼82%) were selected for transport assays. We found that vesicles containing TgNST1 transported UDP-GlcNAc and UDP-GalNAc about 6-fold (UDP-GlcNAc) and 90-fold (UDP-GalNAc) more efficiently than vesicles from yeast transformed with the vector alone (Figure 1B). These results confirm that TgNST1 is a *bona fide* NST, and the high UDP-GalNAc transport activity of TgNST1 is congruent with the hypothesis that it could be involved in the glycosylation of cyst wall components that react with lectins specific for GalNaAc and GlcNAc.

### Deletion of *TgNST1* in type II parasites

**Figure 2:**
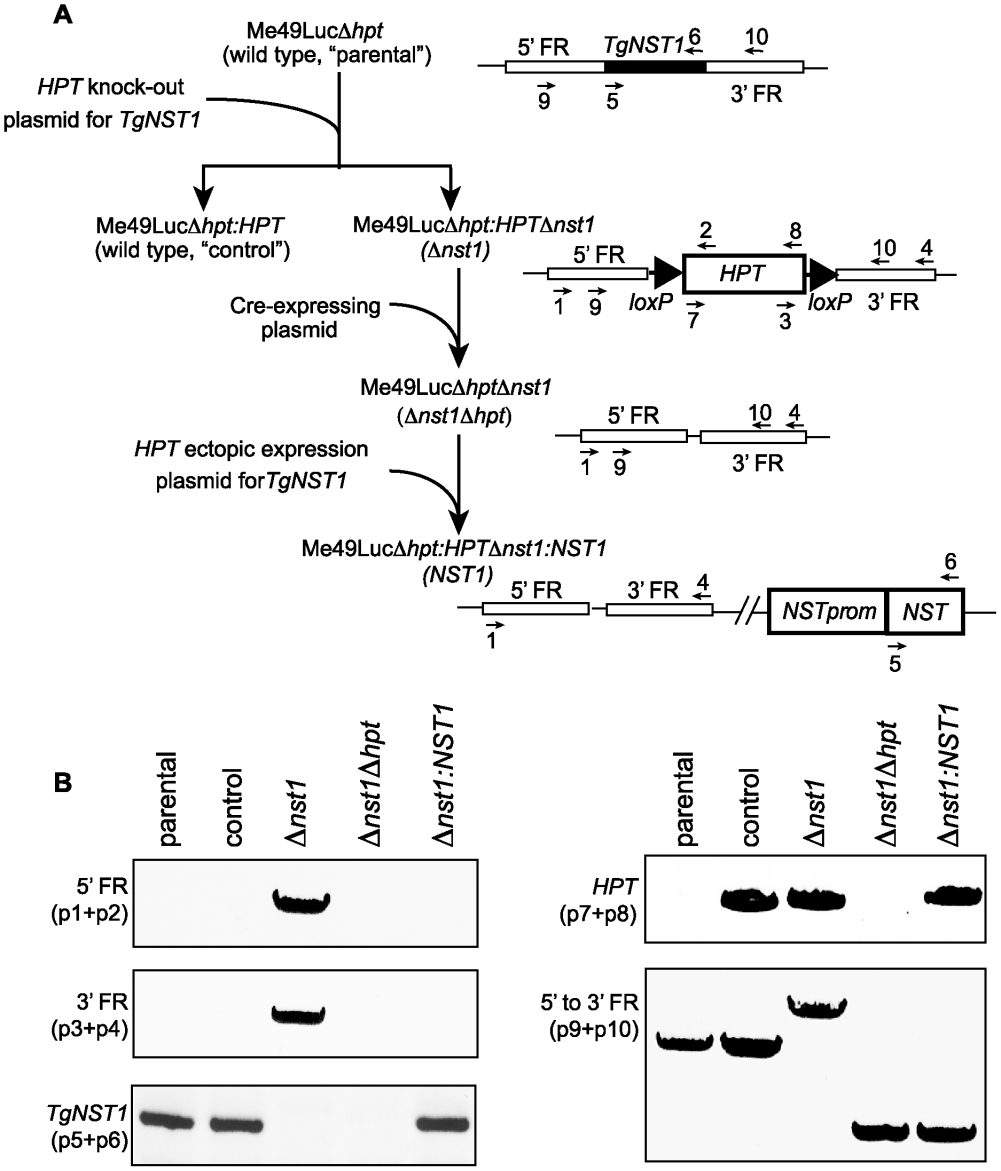
Design and verification of the strain deficient in NST1 and the corresponding complemented strain. A) Workflow of the generation of the wild type, Δ*nst1* and *Δnst1:NST1* strains. 5′- and 3′-FR indicates the 5′- and 3′-flanking regions of the TgNST1 gene used for the homologous recombination required for deletion of the gene. Primers used for PCR analysis are numbered. B) The engineered strains of panel A were analyzed by PCR using primers that specifically amplify the 5′- or 3′-flanking regions (primers 1+2 and 3+4, respectively), the NST1 coding region (primers 5+6), the HPT cassette (primers 7+8), or the 5′- to 3′- flanking regions (primers 9+10).

Since type II *Toxoplasma* parasites are less virulent and establish a chronic infection in mice, we chose this genetic background for engineering a knockout line. Specifically, we used Me49Δ*hpt:Luc*, a type II strain generated by replacing the *hypoxanthine-xanthine-guanine phosphoribosyltransferase* (*HPT*) locus by the gene encoding the reporter enzyme firefly luciferase driven from a tubulin promoter [15]. Figure 2A shows the workflow for the generation of the different strains. We first deleted the *TgNST1* gene by homologous recombination using the pTKO2c vector in which an *HPT* cassette is flanked by the corresponding mutant *loxP66* and *loxP71* sites. Among parasites stably expressing the *HPT* cassette from the knockout plasmid construct, we cloned Δ*nst1* parasites with the correct insertion of the construct and wild type (WT) parasites in which the *HPT* gene integrated ectopically in the genome without replacing the *TgNST1* coding region by genomic PCR (Figure 2B). After removing the *HPT* selector cassette by Cre-mediated recombination, we created a *Δnst1:NST1* complemented strain using the pTKO2c vector encoding the *TgNST1* promoter and coding region to ectopically express this gene in the knockout strain. Figure 2B, left top and middle panels show amplification of the 5′- and 3′-flanking regions but not the *TgNST1*-coding region for the Δ*nst1* strain. As expected, this latter region is amplified using genomic DNA from the parental, control (wild type) and *Δnst1:NST1* strains. Figure 2B, top right panel denotes the presence of the *HPT* gene integrated in the genome of the control (wild type), Δ*nst1* and *Δnst1:NST1* strains. Additionally, this panel indicates the removal of the *HPT* cassette by Cre-mediated recombination. Finally, figure 2B, bottom right and left panels together show the ectopic integration of the *TgNST1* gene in the *Δnst1:NST1* strain. Deletion of *TgNST1* had no detectable effect on the growth rate *in vitro* as determined by the perimeter of the plaque formed 8 days post-infection of HFF monolayers (data not shown).

### Deletion of *TgNST1* affects both tachyzoite and bradyzoite glycosylation

**Figure 3:**
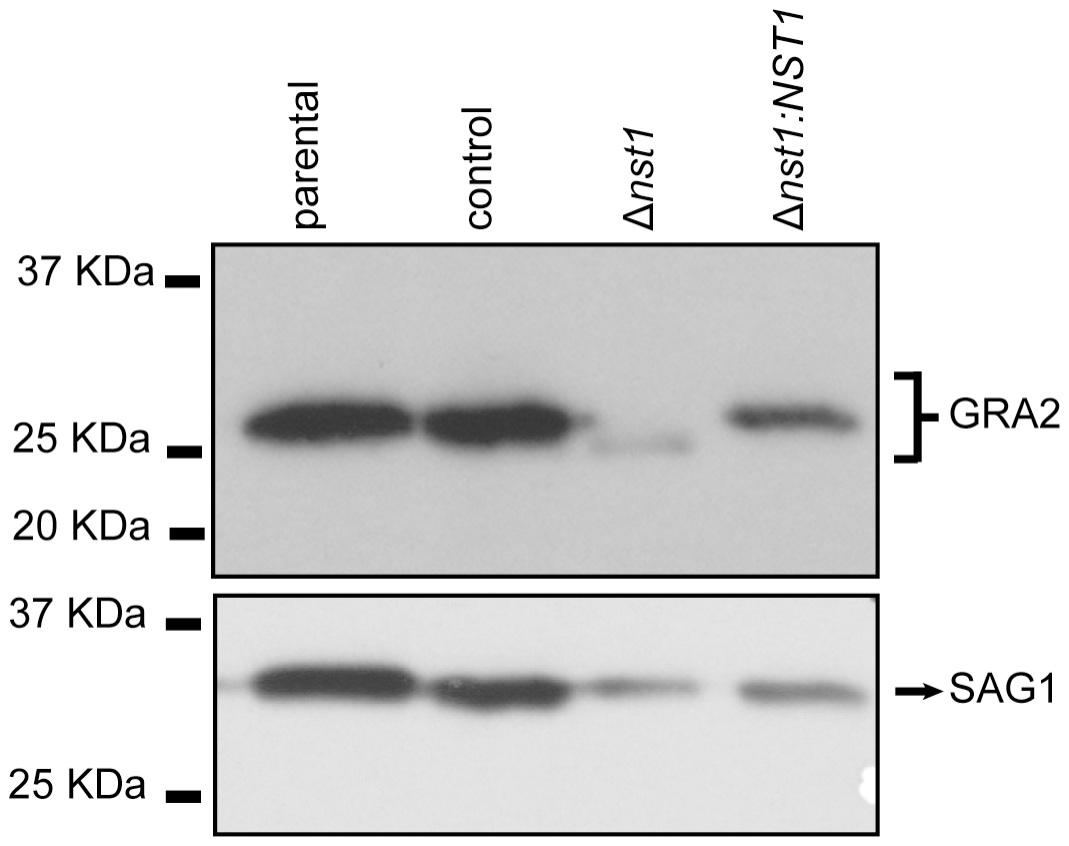
Deletion of *TgNST1* affects GRA2 mobility in a SDS-PAGE. Total parasite extracts from parental, control, *Δnst1* and *Δnst1:NST1* strains were separated by SDS-PAGE and proteins transferred to PVDF membrane. The mobility of GRA2 protein was analyzed using a mouse anti-GRA2 antibody (upper panel). The blot was stripped and reprobed with antibodies to SAG1 as a loading control (lower panel).

Expression data posted by several groups on ToxoDB v7.2 indicate that *TgNST1* is transcribed at substantial levels in both the tachyzoite and bradyzoite stages, with similar levels of transcript abundance in the two stages (∼60^th^–70^th^ percentile of all genes). We therefore began by assessing the effect of *TgNST1* deletion on tachyzoite glycoproteins by analyzing the dense granule protein GRA2, as this has been previously reported to be *O*-modified with GalNac residues [16], a process that should be disrupted by loss of TgNST1 activity in the mutant. Figure 3A shows that the GRA2 protein expressed by *Δnst1* parasites migrates at a faster rate in SDS-PAGE compared to protein from the parental and control wild type strains, consistent with a loss of some or all the sugar side-chains on the *Δnst1-*derived GRA2. The ectopic introduction of TgNST1 in the *Δnst1:NST1* strain resulted in a restoration of GRA2's mobility to that seen for WT (Figure 3). Although we cannot exclude the possibility that the mobility difference is due to some other post-translational modification, this result suggests that the *Δnst1* deletion affects GRA2 glycosylation in tachyzoites, and is consistent with the TgNST1 specificity determined biochemically above. We did not attempt to quantify the overall expression levels of GRA2 in the different strains but the data shown in Figure 3 suggest that there may be some small effect on the synthesis, stability or trafficking of this protein.

**Figure 4:**
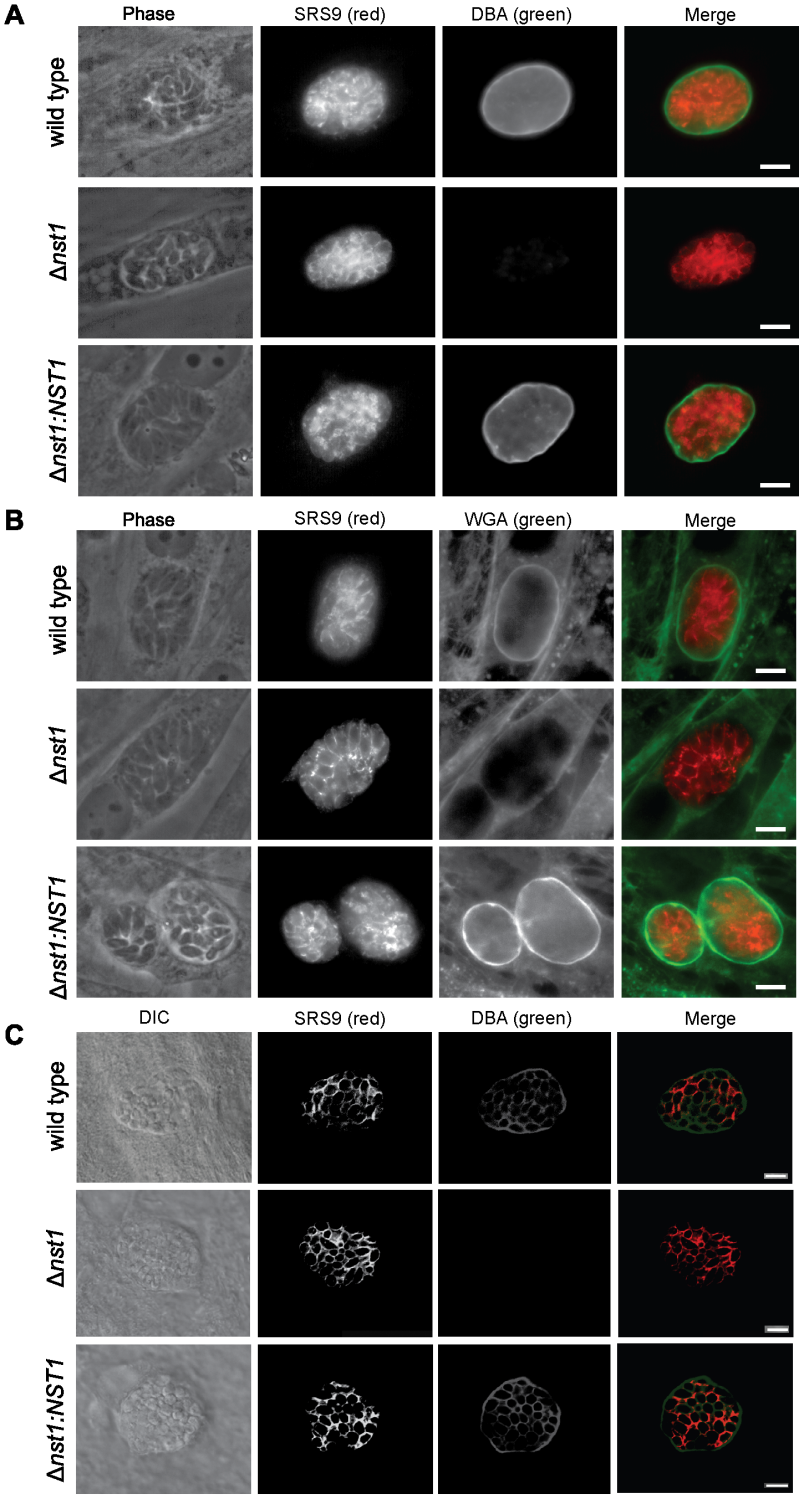
*TgNST1* deletion interferes with recognition by the DBA and WGA lectins. WT, *Δnst1*, or *Δnst1:NST1* parasites were grown under bradyzoite-inducing conditions for 4 days *in vitro*, fixed in formaldehyde, permeabilized and stained using the FITC-conjugated lectins DBA (A) or WGA (B). Antibodies against the bradyzoite-specific antigen SRS9 and Alexa 647-conjugated goat anti-rabbit antisera (pseudocolor red) were use to identify bradyzoites within cysts. Scale bar represents 10 µm. C) Mice infected with the WT, *Δnst1* and *Δnst1:NST1* strains were sacrificed 3 weeks post-infection, perfused with heparine/saline and brains were drop-fixed in paraformaldehyde, sucrose-embedded and cryosectioned for immunohistochemistry. 40 µm sections were stained using the FITC-DBA lectin and antibody against the bradyzoite-protein SRS9, as in parts A and B. 1 µm slice images were taken using a Zeiss confocal microscope and a single slice is shown. Scale bar represents 10 µm.

To evaluate the role of TgNST1 in the glycosylation of bradyozoite cyst wall components, we performed immunofluorescence labeling of *in vitro* tissue cysts using the lectins DBA and WGA. *In vitro* tachyzoite cultures from the WT, Δ*nst1* and *Δnst1:NST1* strains were induced to encyst by alkaline treatment for 4 days. Figures 4A and 4B show the lack of DBA- and WGA-staining of *in vitro* Δ*nst1* cysts, in contrast to the bright staining of WT cysts. Lectin staining was fully restored in the *Δnst1* background when the TgNST1 activity is complemented by ectopic expression. Furthermore, confocal images of DBA-staining on *in vivo* cysts recovered from brains of mice infected with these strains showed the same phenotype (Figure 4C). This confocal image also revealed clear TgNST1-dependent staining between or, perhaps, at the periphery of each parasite, in addition to the cyst wall. This pattern, which is likely due to staining of *O*-glycosylated GRA2 and probably other secreted proteins, was less apparent in the epifluorescence used for visualizing the *in vitro* tissue cysts (Figures 4A and 4B) but this could be due to limitations of epifluorescence rather than a real difference. Importantly, antibodies that recognize the bradyzoite-specific antigen SRS9 efficiently stained the surfaces of *in vitro* and *in vivo* WT, Δ*nst1*, and *Δnst1:NST1* encysted bradyzoites, demonstrating that TgNST1 activity is not required for efficient tachyzoite-to-bradyozoite conversion (Figure 4A–C).

**Figure 5:**
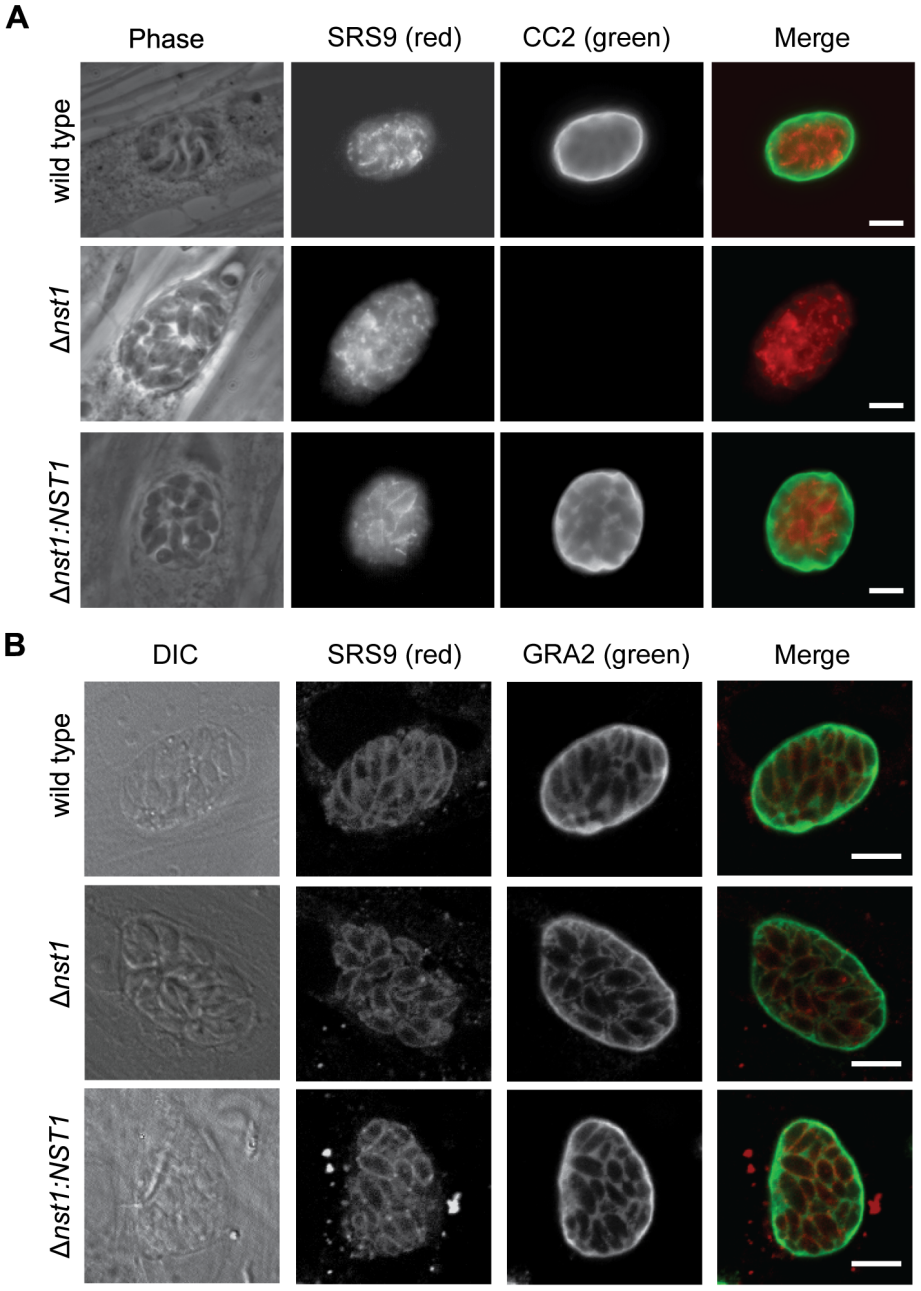
*TgNST1* deletion interferes with CC2 antibody recognition but does not affect GRA2 secretion. *In vitro* cysts were fixed in formaldehyde, permeabilized and stained using the rat monoclonal antibody CC2, specific for the tissue cyst wall (A) or the mouse monoclonal antibody GRA2, specific for the cyst wall and intravacuolar material surrounding the parasites (B). Both these antibodies were detected with Alexa-488-conjugated secondary antibodies and visualized by epifluroescence (part A) or confocal (part B) microscopy. Antibodies against the bradyzoite-specific antigen SRS9 and Alexa-647-conjugated goat-anti-rabbit were use to identify bradyzoites within cysts. For confocal images, 1 µm slice images were taken using a Zeiss confocal microscope and a single slice is shown. Scale bar represents 10 µm.

In a complementary experiment, we asked if deletion of *TgNST1* alters the epitopes recognized by the CC2 and 73.18 monoclonal antibodies (mAbs), which have been reported to recognize cyst wall glycoproteins [10], [11]. Figure 5A shows that CC2 antibodies fail to recognize the cyst wall of *in vitro* Δ*nst1* cysts, whereas binding is restored in cysts complemented with TgNST1. Similar results were obtained with the MAb 73.18 antibody (data not shown). These results suggest that the deletion of *TgNST1* specifically impairs the glycosylation of cyst wall components without affecting the *in vitro* capability of tachyzoite-to-bradyzoite differentiation.

### *TgNST1* deletion does not affect protein secretion to the cyst wall

Thus far, we have shown that TgNST1 activity is required for proper glycosylation of parasite cyst wall components *in vitro* and *in vivo*. However, these experiments could not eliminate the possibility that Δ*nst1* parasites are defective in general secretion of proteins to the cyst wall. It has been shown that the inhibition of a CMP-Sialic Acid-transporter, a GDP-Fucose-transporter, or both by RNAi in HeLa cells causes accumulation of glycoconjugates in the Golgi instead of being transported to the plasma membrane [17]. To examine this possibility, we assessed whether the lack of GRA2 glycosylation in the *Δnst1* strain interferes with GRA2 localization to the cyst wall. Figure 5B shows immunofluorescence micrographs of WT, Δ*nst1* and *Δnst1:NST1 in vitro* cysts using anti-GRA2 antibodies, demonstrating that GRA2 is efficiently localized to the cyst wall in WT and Δ*nst1 in vitro* cysts, although these data do not allow for a quantitative assessment. Furthermore, we found that the dense granule protein GRA9 [18] and the recently identified cyst wall protein BPK1 [19] are also efficiently secreted to the cyst wall matrix (data not shown). These results indicate that the defects observed in Δ*nst1* cysts are specific to glycosylation modifications and not to an overall alteration in the localization of cyst wall components.

### TgNST1 activity is not required for virulence during the acute stage of infection in mice

**Figure 6:**
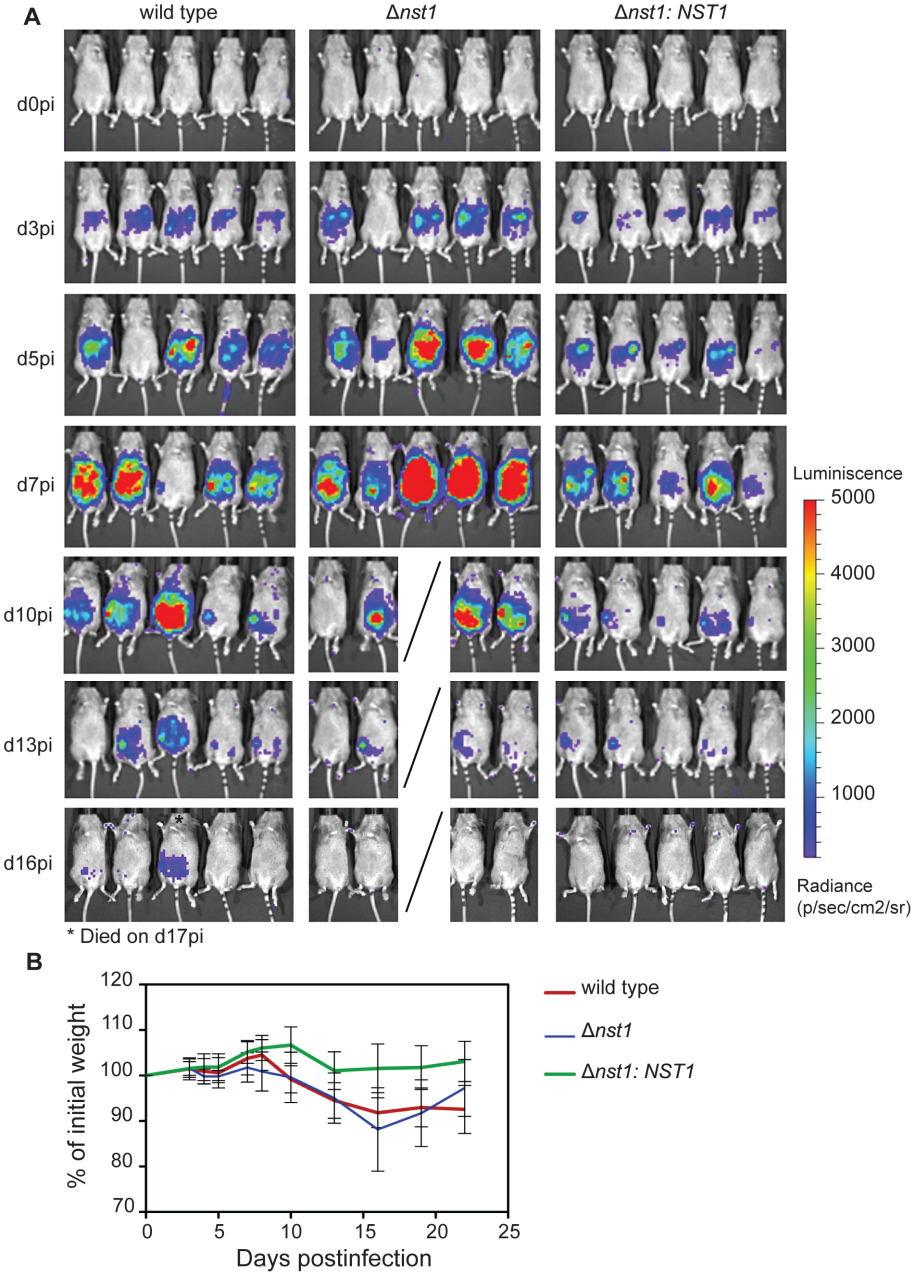
TgNST1 activity is not required for virulence during the acute stage of infection in mice. Mice were infected i.p. with 500 WT, Δ*nst1* or Δ*nst1:NST1* tachyzoites. A) At the indicated days post-infection (pi), animals were injected i.p. with 200 µL of d-luciferin in PBS (150 mg substrate/kg body weight), anesthetized with isoflurane and imaged using the Xenogen IVIS200 charge-couple. Mice were imaged ventrally for 5 min. B) Infected animals shown in panel A were weighed at the indicated days post-infection and loss of weight was plotted as the percentage of the initial weight over time. Weights for animals infected with WT, Δ*nst1* or Δ*nst1:NST1* parasites are shown with green, red and blue lines, respectively.

Having shown that the absence of TgNST1 has a profound effect on cyst wall glycosylation, we next examined the impact of this absence on *Toxoplasma* infection in animals. We intraperitoneally infected CBA/J female mice with 500 WT, Δ*nst1* or Δ*nst1:NST1* tachyzoites and followed the progression of infection by parasite bioluminescence and weight-loss. The dissemination kinetics observed during the first 2 weeks post-infection in the WT and Δ*nst1* strains displayed no significant differences (Figure 6A). While mice infected with the Δ*nst1* strain display a slightly higher abdominal bioluminescence than the other groups, mice were equally able to control the acute proliferation of the three strains as indicated by the lack of detectable photon flux from any region of the body by day 17 pi. By day 10 post-infection, only 1 mouse out of 5 infected with the Δ*nst1* parasites had died and 1 mouse out of 5 infected with WT parasites died by day 17 post-infection, indicating that there is little to no difference in mortality rate during the acute stages of the infection. Furthermore, all mice infected with WT or Δ*nst1* parasites experienced a similar percentage weight loss, with a maximum loss around 2 weeks post-infection (Figure 6B). Hence, TgNST1 activity appears not to be required for virulence in acute infections. Surprisingly, the proliferation rate of the complemented *Δnst1:NST1* strain observed by bioluminescence was somewhat slower than either the parental or the *Δnst1* strains. This is consistent with this strain having a slight growth defect *in vitro* (after 9 days of growth, the plaque perimeter was on average approximately half of wild type plaques, data not shown). The basis for this phenotype is not known but could be due to a deleterious effect of the possible over-expression of TgNST1 in the complemented strain and/or a consequence of a gene disruption at the site of ectopic (non-homologous) integration of the complementing vector. As this result had no bearing on the conclusion that the lack of TgNST1 is without consequence during the acute growth (growth of the control and Δ*nst1* strains were indistinguishable), we did not further pursue the basis of this mild phenotype.

### TgNST1 activity is required for parasite persistence in the brain during chronic infection of mice

**Figure 7:**
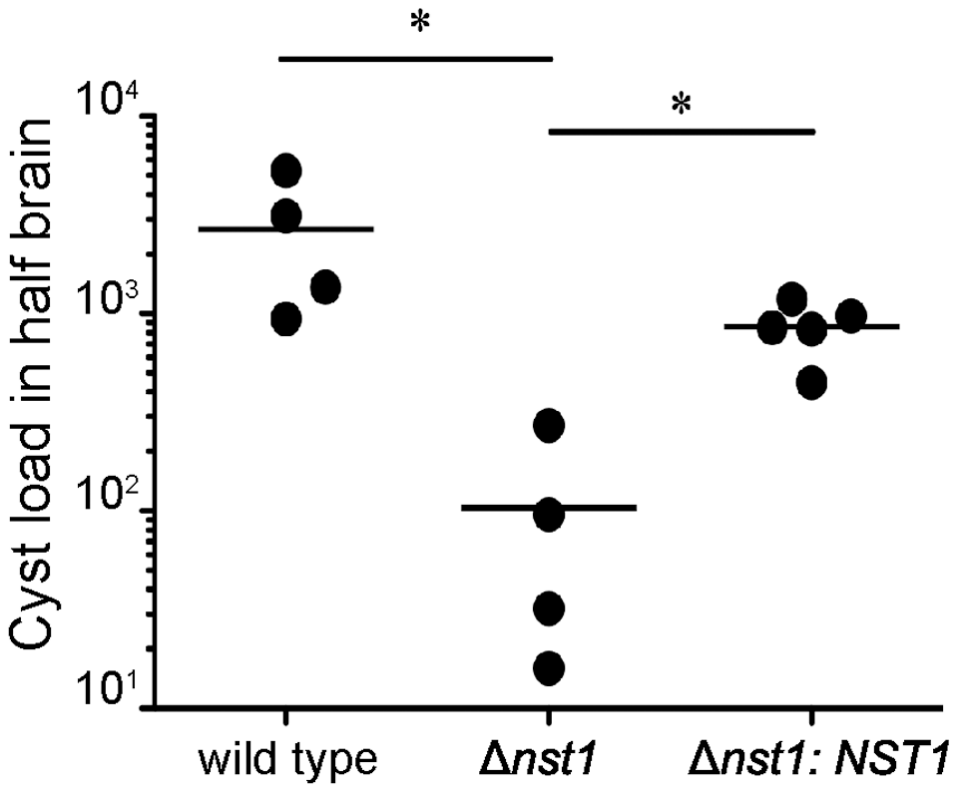
Mice infected with the Δ*nst1* strain show reduced numbers of cysts in the brain. Mice were infected i.p. with 500 WT, Δ*nst1* or Δ*nst1:NST1* tachyzoites. 3 weeks post-infection, animals were sacrificed by perfusion and half the brain was homogenized for cyst enumeration using antibodies against the bradyzoite-specific antigen SRS9 to facilitate detection of cysts. The figure shows representative results from two independent experiments. Horizontal bars indicate the mean for each group. Asterisks indicate statistically significant differences (p = 0.05) between indicated groups.

To determine if the deletion of *TgNST1* affects the formation or persistence of cysts during the chronic stage, infected animals were sacrificed 21 days post-infection and brains were collected. The right hemispheres were individually homogenized and cyst loads were determined by immunofluorescence using antibodies against the bradyzoite-specific antigen SRS9. The results showed that mice infected with the Δ*nst1* strain harbor dramatically lower cyst loads in the brain compared to mice infected with the WT strain (Figure 7). Introduction of the *TgNST1* gene almost fully restored the brain cyst load (the somewhat lower level relative to the parental control was not statistically significant), despite the fact that the acute stages of infection with this strain may be somewhat attenuated. This confirms that the reduction in cyst count in the *Δnst1* strain is due to a lack of TgNST1 activity.

### TgNST1 activity is not required for parasite tropism to the brain

**Figure 8:**
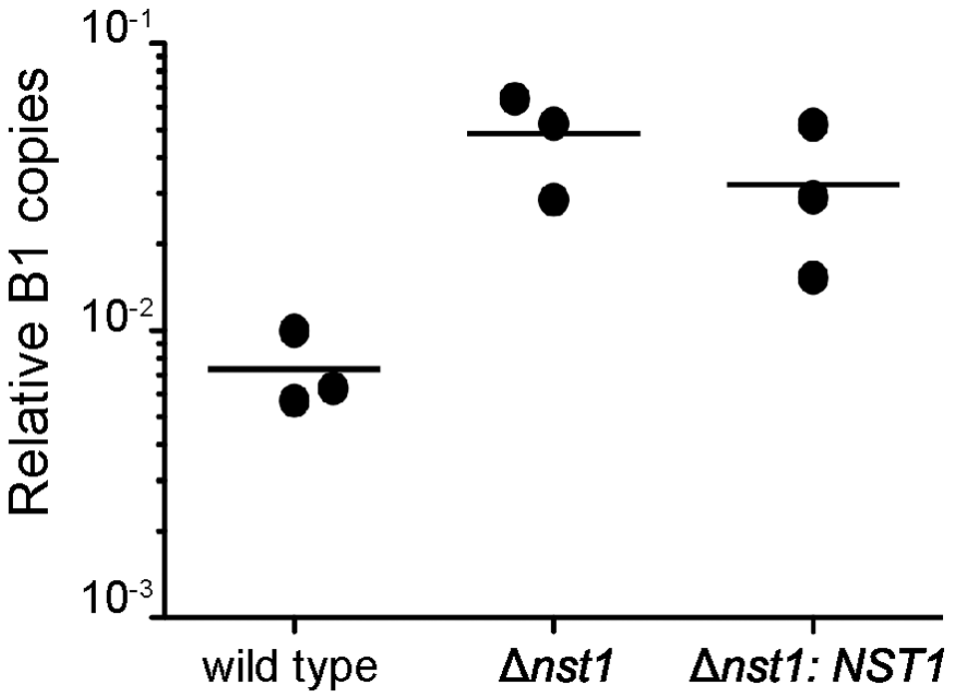
Δ*nst1* parasites are not defective in tropism to the brain. Mice were infected i.p. with 500 WT, Δ*nst1* or Δ*nst1:NST1* tachyzoites.12 days post-infection, animals were sacrificed and the posterior left quarter of the brain was used to quantify the number of parasites present by real-time quantitative PCR detection of *Toxoplasma*'s B1 gene normalized to the detection levels of mouse actin (ACT1).

The significant decrease in the brain cyst counts in Δ*nst1* could be due to defects in this strain's tropism for the brain, their *in vivo* encystation capabilities and/or the ability of tissue cysts containing them to persist once formed. The *in vivo* bioluminescence experiments showed that the acute, tachyzoite stages of the wild type, *Δnst1* mutant and complemented strains are effectively controlled by two weeks post-infection (Figure 6A), by which time it would be expected that some number of the parasites have differentiated to bradyzoites and initiated encystation. To examine whether Δ*nst1* parasites are deficient in their ability to reach the brain at early times during the infection, we intraperitoneally infected mice and evaluated the presence of parasites in the brain by genomic real-time quantitative PCR. This time point was chosen based on previous studies that the acute infection in the brain typically begins to peak about this time [20]. The qPCR results shown in Figure 8 demonstrate that Δ*nst1* parasites reach the brains of infected mice with comparable or even better efficiency than wild type and Δ*nst1:NST1* strains. The limited analysis, however, precluded any more quantitative conclusions. Hence, these results demonstrate that Δ*nst1* parasites are not defective in tropism to the host brain during the acute stages of infection. These results did not allow us to discriminate between the low cyst number being due to a defect in the initial formation vs. subsequent survival of the cysts; nevertheless, the fact that the cyst load was dramatically lower just 3 weeks into the infection indicates that the defect manifests very early in the chronic stages of the infection.

## Discussion

Here we have identified and characterized a *Toxoplasma* nucleotide-sugar transporter, TgNST1, that is required for the synthesis of cyst wall glycoconjugates and whose deletion results in lower cyst numbers during chronic infection. Our results *in vitro* show that *Δnst1* parasites are not defective in the formation of otherwise normal cyst walls, as we demonstrated that the glycosylated cyst wall protein GRA2 and the cyst wall proteins GRA9 and BPK1 are efficiently secreted and incorporated into the cyst matrix surrounding such parasites. This suggests that the glycan moieties of *Toxoplasma* tissue cysts (protein or otherwise) play a particular role in parasite persistence and the maintenance of a chronic infection. Our results do not address the identities of the key glycan moieties responsible for this phenotype, a complete inventory of which has not been determined for bradyzoites or the cyst wall within which they reside. We cannot exclude, therefore, the possibility that NST1 is involved in modifications involving other glycan residues not tested here. Based on glycoconjugates identified in tachyzoites, however, there are four types of molecules that could be involved: glycosylphosphatidylinositols (GPIs), *N*-glycans, *O*-glycans and glycosphingolipids. *Toxoplasma* GPIs consists of a conserved core phosphatidylinositol-lipid structure linked to a glucosamine and three mannose residues. In addition, *Toxoplasma* GPIs can be modified by a glucose-α1-4-*N*-Acetylglucosamine side chain attached to the first mannose. Although we did not specifically examine GPI biosynthesis in the *Δnst1* parasites, we saw no differences in the surface staining with antibodies to abundant GPI-anchored proteins like SRS9 (Figures 4 and 5), arguing against a role for TgNST1 in GPI biosynthesis. Such is also suggested from the fact the GPIs are important in the pathogenesis of the acute stage of an infection with *Toxoplasma* [21], [22] and we saw no such phenotype with the *Δnst1* parasites. While studies of the topology of GPI biosynthesis in *Toxoplasma* indicate that most of the GPI intermediates, including those with a side chain modification, localize to the cytosolic phase of the ER, these results don't completely rule out their lumenal synthesis and thus the requirement of a nucleotide-sugar transporter [23]. A thorough analysis of GPI structures in Δ*nst1* parasites compared to wild type will be needed to definitively determine if NST1 has a role in GPI biosynthesis.

Data have been obtained to suggest that, although not common, *N*-glycan modifications of proteins play important roles in *Toxoplasma* motility and invasion [24]. However, *Toxoplasma* only possesses enzymes for the biosynthesis of truncated Glc_3_Man_5_GlcNAc_2_-PP-Dol and while it seems that parasites can incorporate the complete precursor Glc_3_Man_9_GlcNAc_2_-PP-Dol from the host into its proteins, none of these structures are further modified in the lumen of the ER/GA [25]. Given this and the fact that NSTs are not involved in the synthesis of the core of *N*-linked glycans in any of the many well-studied systems, ranging from yeast to mammals, it seems highly unlikely that an impairment of NST activity will affect *Toxoplasma N-*glycoproteins. We did not specifically address this, however, through examination of any known *N*-linked glycoproteins.

Our data are consistent with the prediction that TgNST1 activity is required for the synthesis of *O*-glycans and/or glycosphingolipids, and that one or more of these molecules are responsible for proper encystation and/or immune evasion *in vivo*. *Toxoplasma* synthesizes glycosphingolipids *de novo* using GalNAc as a sugar precursor [26]. Our assays in yeast showed that TgNST1 is a highly active UDP-GalNAc transporter and so the synthesis of GalNAc-modified glycosphingolipids likely also will be affected by the loss of TgNST1 activity. Whether it is this or *O*-glycosylation that is more important for persistence of tissue cysts cannot be determined at this point but the fact that deletion of TgNST1 completely eliminates the abundant staining with lectins like DBA indicates that an entire class of structures is lost in the *Δnst1* parasites. While further studies will be needed to determine the critical constituents and precise mechanisms involved, the results reported here show that a subset of sugar modifications are dispensable for the acute stages of *Toxoplasma* infection but are crucially important for the chronic and untreatable form of this important parasite.

## Materials and Methods

### Ethics statement

This study was carried out in strict accordance with the Public Health Service Policy on Humane Care and Use of Laboratory Animals and AAALAC accreditation guidelines. The protocol was approved by Stanford University's Administrative Panel on Laboratory Animal Care (Animal Welfare Assurance # A3213-01, protocol # 9478). All efforts were made to minimize suffering.

### Yeast culture and strain generation

The strain PRY225 (ura3-52, lys2-801am, ade2-1020c, his3, leu2, trplΔ1) was grown at 30°C in liquid yeast extract/peptone/dextrose. When required, solid yeast extract/peptone/dextrose media containing 2% Bacto-agar was used. Strains derived from PRY225 transformed with the pG426 URA plasmid were grown at 30°C in synthetic complete medium lacking uracil (SC-URA). The open reading frame of *TgNST1* (TgME9_067380) was amplified using the following primers: 5′-CGACGGGATCCATGACTGGTCGTGGCGCGG-3′ (forward) and 5′-CGTCGGGTACCTCAcgcgtagtccgggacgtcgtacgggtaCGAACGCTTGTCGCACCAC-3′ (reverse; lower case indicates the HA tag sequence). The PCR product was digested with BamHI/KpnI and ligated to the p426GPD vector to obtain plasmid pG426-TgNST1HA for the expression in yeast.

### Radioactive substrates

Radioactive substrates were purchased from American Radiolabeled Chemicals (St. Louis, MO): UDP-[^3^H]GlcNAc (20 Ci/mmol), UDP-[^3^H]GalNAc (20 Ci/mmol).

### Subcellular fractionation

*S. cerevisiae* transformed with pG426 or pG426-TgNST1HA were grown in SCM-URA liquid medium to an A_600_ of 2. Spheroplasts were produced as previously described by using a total of 1 mg of Zymolyase 100 T (Seikagaku America, Rockville, MD) per g of cells in spheroplast buffer (1.4 M sorbitol, 20 mM NaN_3_, 48 mM 2-mercaptoethanol, and 50 mM potassium phosphate, pH 7.5) at 37°C [27]. The spheroplast suspension was centrifuged, the pellet was resuspended in 1.5 X volume of membrane buffer (0.8 M sorbitol, 1 mM EDTA, 10 mM triethylamine) and cells were broken by drawing into a narrow-bore serological pipette followed by homogenization with a Dounce. The suspension was centrifuged successively for 10 min at 2000×g, 8 min at 5000×g, and finally, 45 min at 125,000×g to obtain a pellet fraction enriched in endoplasmic reticulum and Golgi Apparatus-derived vesicles. The final pellet was resuspended in membrane buffer with a Dounce and small aliquots were flash-frozen and stored at −80°C until used.

### Nucleotide sugar transport assay

Transport assays and analysis of the samples were performed as previously described [28]. Briefly, *S. cerevisiae* vesicles (1.0 mg of protein) were incubated at 0 or 30°C in 1 mL of 0.5 M sucrose, 30 mM triethanolamine pH 7.20, 5 mM MgCl_2_, and 5 mM MnCl_2_ with a mix of radioactive and unlabeled nucleotide sugar to be tested. The reaction proceeded for 4 min when it was stopped by addition of 3 mL of cold stop solution (1 M sucrose, 1 mM EDTA). Vesicles were separated from the medium by centrifugation and washed four times with 4 mL of stop solution. The final pellets were resuspended in 1 mL of 4% perchloric acid and incubated on ice for 15 min. The acid-insoluble fraction was separated by centrifugation at 16,000×g for 30 min and the supernatant, containing radioactive nucleotide sugars within the vesicles was counted in 9 mL of scintillation solution. Transport activity is defined as the difference of total radioactive solutes in the vesicle pellet after incubation at 30°C minus the total at 0°C and expressed as pmol of nucleotide sugar/min/mg protein.

### Nucleotidase activity

As previously described, 10 µg of membrane protein was incubated with a mixture of 2 mM CaCl_2_, 200 mM imidazole pH 7.5, 2 mM nucleoside diphosphate (GDP) and with or without 0.1% Triton X-100 in a final volume of 100 µL for 30 min at 30°C [29]. The reaction was stopped by addition of 200 µL of 7.5% SDS. To measure the inorganic phosphate released 700 µL of Ames reagent (0.42% ammonium molybdate in 1N sulfuric acid: 10% ascorbic acid, 6∶1) was added and incubated for 20 min at 45°C. Absorbance was measured at 660 nm.

### *Toxoplasma* culture and *in vitro* differentiation

Human foreskin fibroblasts (HFFs) were cultured in DMEM supplemented with 10% FBS, 100 U/mL penicillin, 100 µg/mL streptomycin, and 2 mM L-glutamine in a 5% CO_2_ incubator (cDMEM). Me49-Luciferase strain parasites were maintained as tachyzoites by passage in HFF monolayers. *In vitro* differentiation of tachyzoites to bradyzoites was performed by switching 4 hour-infected HFFs (moi = 1.0) to HEPES-buffered RPMI 1640 (pH 8.0) supplemented with 1% FBS in an air incubator for at least 4 days.

### Generation of pTKO2c/g plasmids

The pTKO2 plasmid used to engineer the different strains was generated by modification of the previously described pTKO vector [30]. The main distinguishing feature between pTKO and pTKO2 is that the former contains wild type *loxP* sites, whereas the latter contains mutant *loxP66* and *loxP71* sites [31]. The sequences of the mutant *loxP* sites are: *loxP66* [left element] = 5′-TACCGTTCGTATAGCATACATTATACGAAGTTAT-3′; *loxP71* [right element] = 5′-ATAACTTCGTATAGCATACATTATACGAACGGTA-3′. The use of mutant *loxP* sites serves to increase the frequency of Cre recombinase-mediated excision of the *HPT* cassette by preventing re-integration of the excised closed-circular *HPT* cassette containing a single *loxP* site [31]. Furthermore, mutant *loxP* sites resulting from a Cre-mediated recombination event enable a second round of genome engineering using a different pTKO2 plasmid without the risk of recombination at the previously introduced *loxP* site. The resulting pTKO2 plasmid also contains the two multiple cloning sites from pTKO at the 5′ and 3′ ends of the corresponding *loxP* sites. Additionally, a minigene containing the *Toxoplasma GRA2* promoter and mCherry (pTKO2c) or GFP (pTKO2g) open reading frame flanked by the corresponding UTRs was introduced into the backbone of pTKO2. The expression of these fluorescent proteins could be used to assess transfection efficiency and/or to quickly screen for colonies that grow in the presence of mycophenolic acid (MPA) and xanthine (XAN), indicating that the *HPT* cassette is expressed, but are mCherry/GFP-positive or -negative by fluorescence microscopy. The former are typically nonhomologous integrants that have retained the mCherry/GFP minigene. A plasmid map of pTKO2g and its salient features is shown in Figure S1.

### Generation of *Δnst1* and *Δnst1:NST1* parasites

The *TgNST1* gene (TgMe49_067380) was targeted in the Me49Luc strain by homologous recombination using the pTKO2c vector and the *TgNST1* locus was replaced with the *HPT* gene. The ∼2 Kb genomic sequence flanking the *NST1* open reading frame was amplified by PCR using the following primers: 5′-GCGGCGGCCGCCGAACCGGCGGAGGG-3′ and 5′-CGCGCTCGAGGCAGAGACGTGAATGCGGGG-3′ for the 5′-flanking region and 5′-CGCGGCTAGCGCCCATGCAGTGGG-3′ and 5′- CGCGGGGCCCGTCCGATGGGCGTC-3′ for the 3′-flanking region. The 5′-flaking region was cloned into the NotI and XhoI restriction sites and the 3′ flanking region was cloned into the NheI and ApaI restriction sites of pTKO2c. The vector was linearized by digestion with NotI, and 25 µg of plasmid was transfected into the Me49LucΔ*hpt* strain (parental strain) of parasites by electroporation, as described previously [32]. The parasites were allowed to infect HFFs cells and on the following day the medium was replaced with complete DMEM supplemented with 50 µg/mL of MPA and 50 µg/mL of XAN to select for HPT activity. Parasites were syringe-lysed and passed twice into fresh T25 flasks followed by single-cell cloning in 96-well plates. Identification of the control and Δ*nst1* clones was done by PCR for the presence of the *nst1* open reading frame and for the specific recombination of the targeting construct at the *nst1* locus using the following primers: P1) 5′-CCGACGGACTTGGGACC-3′, P2) 5′-ACAGATCGCTCCAACAGCTT, P3) 5′-GCGCACGGCAGTCAGATAAC-3′, P4) 5′-GCCTCACGTAGCGGTTG-3′, P5) 5′-CGGTCGCAGCCGTCCTCG-3′, P6) 5′-GCACCACTCACCGCGAAGCC-3′ (Figure 2). The control clone was selected as a sibling clone of the *Δnst1* clone in which the targeting construct integrated non-specifically somewhere else in the genome leaving the *NST1* locus intact. The *Δnst1Cre* strain was generated to remove the *HPT* gene by transient transfection of the *Δnst1 parasites* with a plasmid encoding Cre recombinase producing a transient, self-limited expression of Cre, sufficient for removal of the *HPT* gene in pTKO2c through recombination at the flanking *loxP* sites. Removal of the *HPT* gene was confirmed by PCR using the primers P7) 5′-AAGCTGTTGGAGCGATCTGT-3′, P8) 5′-GTTATCTGACTGCCGTGCGC-3′ (Figure 2). Parasites were grown in complete DMEM medium supplemented with 6-thioxanthine to select for *Δhpt* parasites. To engineer the complemented strain *Δnst1:NST1*, *Δnst1Δhpt* parasites were transfected by electroporation with the pTKO2c-TgNST1 plasmid containing the *TgNST1* promoter and open reading frame followed by selection in MPA/XAN medium and single-cell cloning by limiting dilution. The pTKO2c-TgNST1 plasmid was generated by PCR amplification of *TgNST1* using the primers 5′- GCGGTGGCGGCCGCATCGAGACCGCGAGTCTGTC-3′ and 5′- CACGGCTCGAGCTAcgcgtagtccgggacgtcgtacgggtaCGAACGCTTGTCGCACCAC-3′ (lower case indicates a HA tag sequence). The purified PCR product was restriction digested with NotI and XhoI and ligated to the corresponding sites in the pTKO2c vector. Primers P9) 5′-CAGCTCGACCCATGTGCG-3′ and P10) 5′-GGCACCACAGCCGTTAACGC-3′ were used to verified that the integration of the *TgNST1* promoter and open reading frame didn't occur at the endogenous locus (Figure 2).

### Protein gel electrophoresis and Western blotting

Tachyzoites were obtained from infected cells by syringe-lysis. After washing in PBS the pellet was resuspended in SDS-PAGE sample buffer. Samples were boiled for 5 min, separated by SDS-PAGE and transferred to a PVDF membrane. Membranes were blocked in PBS/0.1% Tween/5% nonfat dry milk and incubated with mouse anti-GRA2 or rabbit anti-SAG1 followed by peroxidase-conjugated goat anti-rabbit IgG and goat anti-mouse. Detection was done by ECL chemiluminescence reagents.

### Immunofluorescence microscopy

*In vitro* cysts grown in coverslips were fixed in 2.5% formaldehyde in PBS for 20 min at 37°C. After washing twice with PBS, samples were blocked with PBS/3% BSA for 1 hour at room temperature. Permeabilization was performed with PBS/0.2% Triton-X100/3% BSA for 20 min at room temperature followed by a wash in PBS. The following primary antibodies were incubated for 1 hour at room temperature, diluted as indicated in PBS/0.01% Triton-X100/3% BSA: rabbit anti-SRS9 (1∶2000), rat anti-CC2 (1∶50) [11], mouse anti-GRA2 (1∶1000). Coverslips were washed with PBS for 5 min for a total of 5 washes. The corresponding secondary antibodies from Molecular Probes were used at a 1∶2000 dilution in PBS/0.01% Triton-X100/3% BSA and incubated for 1 hour at room temperature. After washing with PBS 5 times for 5 min each time, samples were mounted using Vectashield from Vector Laboratories. Images were taken using the ImageJ software and a 35 mm digital camera (model C4742-95; Hamamatsu) connected to an upright fluorescence microscope (model BX60; magnification, ×1000; Olympus). Confocal images were taken with a Zeiss LSM700 scanning confocal.

### Mouse infection

Tachyzoites from the control, *Δnst1* and *Δnst1:NST1* strains were release form infected HFFs by syringe-lysis. Parasites were washed in PBS and counted with a hemocytometer. Groups of 5 seven-week-old CBA/J female mice (The Jackson Laboratory, stock # 000656) were injected i.p. with 500 tachyzoites in 200 µL of PBS. We considered ≥3 weeks postinfection as the chronic phase of infection. Bioluminescence from all animals was imaged regularly using the Xenogen IVIS200. Mice were injected i.p. with 200 µL of d-luciferin in PBS (150 mg substrate/kg body weight) and anesthetized with isoflurane. Imaging was started 10 min after the substrate was given. Mice were imaged ventrally for 5 min.

### Enumeration of cyst loads in the brain and immunohistochemistry

3 weeks post-infection, mice were anesthetized with a ketamine/xylazine cocktail (24 mg/mL and 48 mg/mL respectively) and intracardiacally perfused with heparin (10 U/mL) in a 0.9% saline solution. Brains were collected and divided into 3 samples as follows: the right frontal quarter was drop-fixed in 4% paraformaldehyde for 24 hours, cryoprotected in 30% sucrose in 1 X PBS, and stored in 30% sucrose at 4°C until sectioned [33], the right posterior quarter was flash-frozen for real-time quantitative PCR experiments and the left hemisphere was immediately processed for cyst count by homogenizing them through 100 µm cell strainers. The homogenate was then washed in 25 mL of PBS/3% FBS and resuspended in 10 mL of PBS/3% FBS. 5 mL of each sample was fixed in 4% formaldehyde/PBS for 1 hour followed by blocking in PBS/3% FBS for 30 min at room temperature. After permeabilization with PBS/0.2% Triton-X100/3% FBS, homogenates were incubated with rabbit anti-SRS9 (diluted 1∶2000) in PBS/0.01% Triton-X100/3% FBS for 1 hour at room temperature. After washing twice with PBS, samples were incubated with Alexa-488 goat anti-rabbit 1∶200 and DBA-fluorescein 1∶250 in PBS/0.01% Triton-X100/3% FBS overnight at 4°C. Homogenates were washed 3 times with 10 mL of PBS and resuspended in a final volume of 2 mL. 50 µl aliquots were seeded in a flat-bottom 96-well plate and cysts were counted using an inverted fluorescence microscope (model eclipse TE300, 20×, Nikon). Free-floating sagital sections (40 µm) were cut on a sliding freezing microtome (Microm HM 430) and stored at 4°C in cryoprotective medium (0.05 M sodium phosphate buffer containing 30% glycerol and 30% ethylene glycol). Sections were selected at random and blocked overnight with TBS/3% goat serum/0.3% Triton-X100 followed by incubation with anti-SRS9 (diluted 1∶2500) in TBS/1% goat serum/0.3% Triton-X 100. After washing with TBS, samples were incubated with Alexa-488 goat anti-rabbit 1∶200 and DBA-fluorescein 1∶250 in TBS/0.1% goat serum/0.3% Triton-X 100. Brain sections were mounted on slides using Vectashield Hardmount and viewed on a Zeiss LSM700 scanning confocal.

### Real-time quantitative PCR

Mice infected with the control, Δ*nst1* and Δ*nst1:NST1* strains were sacrificed 12 days postinfection by perfusion. The right posterior quarters of the brains were flash-frozen and stored at −80°C until used. Genomic DNA was prepared using the DNeasy blood & tissue kit from Qiagen according to the instructions of the manufacturer. Real-time quantitative PCR was performed using the B1 primers (5′-TCCCCTCTGCTGGCGAAAAGT-3′ and 5′-AGCGTTCGTGGTCAACTATCGATTG-3) [34]. Samples were processed on a real-time detection system (Mx 3000P, Stratagene) using rtTh polymerase (Applied Biosystems) and SYBR-Green. Parasite DNA levels were normalized using the gene for mouse actin (ACT1) as a normalization control (i.e., to normalize to a constant amount of mouse tissue). Results were expressed as the fold change using the formula 2^−Δ^ *CT*, where Δ*CT* = threshold cycle (*CT*) of actin − *CT* of the target gene (B1).

### Plots and statistics

Results were plotted using Graph Pad Prism software and student's t-test was performed to compare parasite loads in different experimental groups.

## Supporting Information

Figure S1 **Plasmid map and salient features of pTKO2g.** pTKO2g was generated by replacing the wild type *loxP* sites in pTKO with the mutant *loxP66* and *loxP71* sites flanked by 5′ and 3′ multiple cloning sites (MCS) as indicated. The ampicillin resistance (AmpR) and the *HPT* (HXGPRT) cassettes allow for propagation in bacteria and selection of *Toxoplasma* transformants, respectively. The pTKOg plasmid also contains a *Toxoplasma* GRA2 promoter driving the expression of the GFP minigene.
(TIF)
