# Supplementary figures and images for: A Nucleotide Sugar Transporter Involved in Glycosylation of the Toxoplasma Tissue Cyst Wall Is Required for Efficient Persistence of Bradyzoites
Source: PLoS Pathog. 2013 May 2;9(5):e1003331. doi: 10.1371/journal.ppat.1003331 (PMC3642066; doi:10.1371/journal.ppat.1003331)

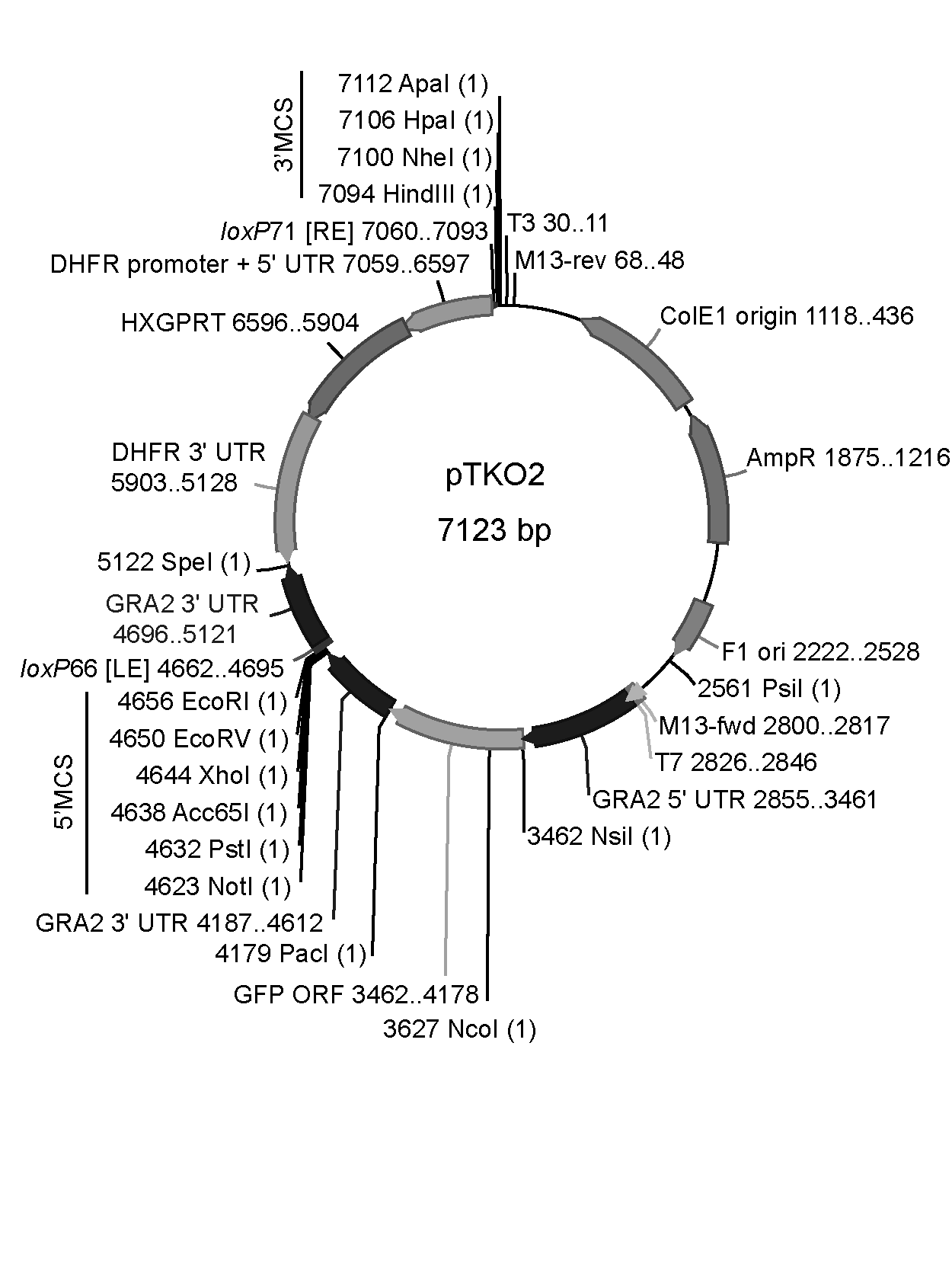

Supplement: Figure S1 — Plasmid map and salient features of pTKO2g. pTKO2g was generated by replacing the wild type loxP sites in pTKO with the mutant loxP66 and loxP71 sites flanked by 5′ and 3′ multiple cloning sites (MCS) as indicated. The ampicillin resistance (AmpR) and the HPT (HXGPRT) cassettes allow for propagation in bacteria and selection of Toxoplasma transformants, respectively. The pTKOg plasmid also contains a Toxoplasma GRA2 promoter driving the expression of the GFP minigene. (TIF) [file ppat.1003331.s001.tif]
